# Prevalence and Diversity of *Staphylococcus aureus* and Staphylococcal Enterotoxins in Raw Milk From Northern Portugal

**DOI:** 10.3389/fmicb.2022.846653

**Published:** 2022-03-22

**Authors:** Ricardo Oliveira, Eva Pinho, Gonçalo Almeida, Nuno F. Azevedo, Carina Almeida

**Affiliations:** ^1^I.P – National Institute for Agrarian and Veterinarian Research (INIAV), Vairão, Portugal; ^2^Laboratory for Process Engineering, Environment, Biotechnology and Energy (LEPABE), Faculty of Engineering, University of Porto, Porto, Portugal; ^3^Associate Laboratory in Chemical Engineering (ALiCE), Faculty of Engineering, University of Porto, Porto, Portugal; ^4^Center for Study in Animal Science (CECA), ICETA, University of Porto, Porto, Portugal; ^5^Centre of Biological Engineering (CEB), University of Minho, Braga, Portugal

**Keywords:** *Staphylococcus aureus*, raw milk, staphylococcal enterotoxins, MRSA, spa typing

## Abstract

*Staphylococcus aureus* and staphylococcal enterotoxins are a serious public health concern associated with hospital and community-acquired illnesses. Dairy animals frequently shed *S. aureus* into the milk supply which can lead to food poisoning in humans. This study aims to investigate the prevalence and genetic diversity of *S. aureus* and staphylococcal enterotoxins in raw milk from the main dairy region of mainland Portugal. *S. aureus* was found in 53.0% (95% CI: 40.6–65.4%) of 100 raw cow’s milk samples collected from bulk cooling tanks. The highest contamination level was 3.4 log10 CFU.mL^–1^, and in some samples more than one *S. aureus* strain was identified. Staphylococcal enterotoxins (SEA-SEE) were detected in one sample. Spa typing revealed 62 distinct *S. aureus* isolates, being t529 (17.7%, 95% CI: 8.2–27.3%) and t1403 (16.1%, 95% CI: 7.0–25.3%) the predominant types, commonly associated with livestock infection or carriage. The antimicrobial susceptibility test showed that 35.5% of the *S. aureus* isolates were resistant to at least one antimicrobial agent, with resistance to penicillin being the highest (32.3%, 95% CI: 20.6–43.9%) followed by tetracycline (24.2%, 95% CI: 13.5–34.9%), ciprofloxacin (16.1%, 95% CI: 7.0–25.3%) and chloramphenicol (16.1%, 95% CI: 7.0–25.3%). Moreover, five isolates (8.1%, 95% CI: 1.3–14.8%) were identified as methicillin-resistant *S. aureus* (MRSA, cefoxitin resistant). Regarding virulence/resistance genes, 46,8% (95% CI: 34.4–59.2%) isolates harbored at least one enterotoxin-encoding gene, and the *seg* gene was the most frequently detected (41.9%, 95% CI: 29.7–54.2%) followed by the *sei* (40.3%, 95% CI: 28.1–52.5%), *sec* (6.5%, 95% CI: 0.3–12.6%), *seh* (4.8%, 95% CI: 0.0–10.2%), and *sea* (1.6%, 95% CI: 0.0–4.7%) genes. Five (8.1%, 95% CI: 1.3–14.8%) non-enterotoxigenic isolates carried the *mecA* gene (corresponding to isolates phenotypically classified as MRSA), and 4.8% (95% CI: 0.0–10.2%) enterotoxigenic strains also had the *tsst-1* gene. Our study confirm that raw milk can be a zoonotic source of *S. aureus*, including enterotoxigenic and MRSA strains. Furthermore, the majority of enterotoxigenic isolates were found to contain genes encoding SEs (SEG, SEH and SEI) not routinely screened. This shows the need for a broader SE screening in food safety control, as well as the relevance of risk mitigation measures to control *S. aureus* transmission along the food chain in Portugal.

## Introduction

Food and water are well-known vectors for the dissemination of zoonotic microorganisms, some of them can be extremely harmful to human health (Gallo et al., 2020). Foodborne diseases are a serious public health concern, associated with losses in productivity and high medical expenses every year (Garcia et al., 2020). Milk, as a central food in the human diet is a critical vehicle of both beneficial and pathogenic microorganisms. Several pathogens including *Brucella* spp., *Campylobacter* spp., Shiga toxin-producing *Escherichia coli*, *Listeria monocytogenes*, *Mycobacterium* spp., *Salmonella* spp., and also bacterial toxins have been associated with milk-borne diseases (Dhanashekar et al., 2012). Furthermore, there has been a drastic increase in antimicrobial resistance among zoonotic pathogens. Thus, food surveillance is a major concern for the food industry and for public health (Pérez-Rodríguez and Mercanoglu Taban, 2019).

*Staphylococcus aureus* is ubiquitous in the environment and a major cause of bovine mastitis. Thus, milk is a common source of contamination for the dairy supply chain, the environment, as well as for final consumers (Rola et al., 2016). In the European Union, 43 foodborne outbreaks (FBO), 402 cases and 32 hospitalizations associated to this pathogen were reported in 2020, according to the recent European Food Safety Authority report (European Food Safety Authority and European Centre for Disease Prevention and Control, 2021b). Typically, staphylococcal food poisoning (SFP) are caused by the ingestion of food contaminated with preformed staphylococcal enterotoxins (SEs) produced by coagulase-positive *staphylococci* (CPS) (Argudín et al., 2010). There are 24 SEs and enterotoxin like (SE*l*-) toxins currently identified, including the classical (SEA-SEE) and the newer (SEG-SElY), which are encoded on different pathogenicity islands (Fisher et al., 2018). Most SFP outbreaks are reported in countries where consumption of unpasteurized milk cheeses is common, such as France and Italy. Additionally, raw milk vending machines and traditionally made food products, such as cheese manufactured at local dairy farms, are becoming more popular throughout Europe, increasing the risk of SFP (Rola et al., 2016).

*Staphylococcus aureus* can also produce several other extracellular virulent proteins such as toxic shock syndrome toxin 1 (TSST-1), Panton-Valentine Leukocidin (PVL), hemolysins, and coagulase. These proteins can contribute to a broad spectrum of pathologies beyond food poisoning that can range from toxin-mediated syndromes to fatal systemic diseases (Chambers and DeLeo, 2009). Moreover, this pathogen is a well-known example of acquired resistance to multiple antibiotics. Methicillin-resistant *Staphylococcus aureus* (MRSA) are of particular concern to human health because it is virtually resistant to all available β-lactam antibiotics and represent a significant cause of morbidity and mortality throughout the world. These have often been found outside the health environment, including in farm animals (van Duijkeren et al., 2014; Wu et al., 2018). Surveillance of raw milk is therefore essential for a better understanding of the risk factors along the milk food chain and to guarantee public health safety.

In Portugal, data on *S. aureus* circulating in raw milk are scarce. Pereira et al. (2009) characterized several *S. aureus* isolates from different foods in Portugal, including a limited number of raw milk samples, and detected the presence of enterotoxigenic *S. aureus*. Molecular typing can be a powerful tool for improve such epidemiological studies with data about clonal relatedness, genetic diversity, and tracking the spread of pathogens. *S. aureus* protein A (spa) typing is a rapid, affordable, and easy molecular typing method that assigns a classification to *S. aureus* strains from the number/sequence variation in repeats at a specific region of the *spa* gene. It offers excellent discriminatory results to the study of *S. aureus* diversity (Sabat et al., 2013). Molecular typing and characterization of virulence factors are thus an important tool in the control of zoonotic diseases.

The aim of this study was to determine the prevalence and diversity of *S. aureus* and staphylococcal enterotoxins in raw cow’s milk collected from bulk cooling tanks on dairy farms from the main dairy region of mainland Portugal. Genetic determinants associated with enterotoxinogenicity (i.e., SE-encoding genes), antimicrobial resistance (i.e., *mecA* and *mecC* genes) and severe human infections (i.e., *pvl* and *tsst-1* genes) were investigated.

## Materials and Methods

### Study Design and Sampling

Between November 2020 and August 2021, 100 raw cow’s milk samples were collected from bulk cooling tanks of 100 dairy farms located in the “Bacia Leiteira Primária de Entre Douro e Minho.” Only dairy cow farms were included in this study. One-liter samples were collected in sterile labeled screwed top bottles, quickly stored at 4°C and analyzed within 24 h. Farms participation was voluntary and anonymous. Information on sampling and number of milking cows of each dairy farm can be found in Supplementary Table 1.

### Detection of Staphylococcal Enterotoxins

The presence of staphylococcal enterotoxins A, B, C, D, and E on raw milk samples was analyzed by a two-step method: extraction/concentration and toxin detection by enzyme linked fluorescent assay (ELFA) with the VIDAS SET2 test (bioMerieux, Marcy-I’Etoile, France) according to ISO 19020:2017 (International Organization for Standardization [ISO], 2017). Confidence Interval (CI), for proportions (Wald method), were determined considering a 95% CI (critical z value of 1.96), prevalence/frequency values and the sample size.

### Isolation and Identification of *Staphylococcus aureus*

Bulk tank milk samples were analyzed according to ISO 6888–2 (International Organization for Standardization [ISO], 2021) method for the enumeration of coagulase-positive *staphylococci* (CPS). Briefly, 1 mL of serial dilutions of raw milk were plated on Baird-Parker agar with rabbit plasma fibrinogen (bioMerieux). After 48 h of incubation at 37 ± 1°C, the number of colonies displaying a phenotype characteristic for CPS were counted and morphologically different colonies were subcultured on tryptic soya agar (TSA) for further identification.

Then, colonies identified as CPS were confirmed as *S. aureus* by the presence of the *nuc* gene. For this, total DNA of each colony was extracted using the boiling method (95°C for 15 min). The suspensions were centrifuged at 12,000 g for 5 min and supernatants employed as DNA template. The primers used to amplify the *nuc* gene had the sequences forward: 5′-GCGATTGATGGTGATACGGTT-3 and reverse: 5′- AGCCAAGCCTTGACGAACTAAAGC-3, as described by Brakstad et al. (1992). The PCR amplification consisted of a reaction mixture containing 1X Supreme NZYTaq II 2 × Green Master Mix (NZYTech, Portugal), 0.25 μM of each primer and 2 μL of DNA. Thermal cycling reaction conditions used were: initial denaturation at 95°C for 5 min, 35 cycles of denaturation at 94°C for 30 s, annealing for 30 s at 56°C and extension at 72°C for 30 s, and a final extension at 72°C for 5 min. *S. aureus* ATCC 25923 and *S. epidermidis* CECT 231 were used as positive control and negative control, respectively. The PCR products were subjected to electrophoresis at 100 V for 1 h in a 1.5% (w/v) agarose gel, previous stained with GreenSafe Premium (NZYTech), and finally analyzed under UV light. CIs for prevalence of CPS were determined by the Wald method as mentioned in “Detection of Staphylococcal Enterotoxins” section.

### *Spa* Typing

*Spa* typing was performed as described by Shopsin et al. (1999) and Roussel et al. (2015). Briefly, amplification and sequencing of the *spa* gene were performed using the total DNA of each *S. aureus* isolate. The PCR amplification consisted of a reaction mixture containing 1X NZYProof Green Master Mix (NZYTech), 0.2 μM of primers spa-1113f (5′-TAA AGA CGA TCC TTC GGT GAG C-3′) and spa-1514r (5′-CAG CAG TAG TGC CGT TTG CTT-3′) and 2 μL of DNA template (Shopsin et al., 1999). Thermal cycling reaction conditions used were: 95°C for 5 min for the initial denaturation, followed by 35 cycles of denaturation at 94°C for 30 s, annealing for 30 s at 56°C and extension at 72°C for 30 s. A final extension was set at 72°C for 5 min. Amplification was confirmed by electrophoresis on a 2% (w/v) agarose gel at 100 V for 1 h, previous stained with GreenSafe Premium (NZYTech). Both strands were then purified and sequenced by I3S genomics platform (Porto, Portugal). The sequences were analyzed using automated workflow provided by BioNumerics software (bioMérieux) which analyze raw sequencer trace files and assign the repeat codes and spa types in connection to SeqNet/Ridom Spa Server1. CIs for prevalence of spa types were determined by the Wald method as mentioned in “Detection of Staphylococcal Enterotoxins” section.

### Antimicrobial Susceptibility Test

Antimicrobial susceptibility profile for the *S. aureus* isolates was determined by the Kirby–Bauer disk diffusion method and interpreted according to the criteria of the Clinical and Laboratory Standards Institute guidelines (Clinical and Laboratory Standards Institute [CLSI], 2020, 2021). Briefly, a colony suspension of each *S. aureus* isolate was resuspended in saline solution at 0.5 McFarland standard. The suspension was streaked on Muller-Hinton Agar (Oxoid, Basingstoke, United Kingdom) and allowed to dry. Then, the antibiotic disks were placed on the medium and incubated at 37°C for 16–18h. The incubation time was extended to 24 h for cefoxitin, which was used as a surrogate test for methicillin resistance. After the appropriate incubation time, the zones of inhibition were measured and interpreted as sensitive (S), intermediate (I), and resistant (R). The following antimicrobials agents were used: penicillin G (PG, 10 IU), cefoxitin (FOX, 30 μg), ceftaroline (CPT, 5 μg), cefoperazone (CFP, 30 μg), ceftiofur (EFT, 30 μg), tetracycline (TE, 30 μg), chloramphenicol (C, 30 μg), gentamicin (CN, 10 μg), trimethoprim-sulfamethoxazole (SXT, 1.25/23.75 μg), trimethoprim (TM, 5 μg), sulfonamides (S, 300 μg), erythromycin (E, 15 μg), ciprofloxacin (CIP, 5 μg), clindamycin (DA, 2 μg), quinupristin-dalfopristin (QD, 15 μg), and linezolid (LZD, 30 μg). *S. aureus* strains ATCC 25923 was used as quality control. CIs for antimicrobial susceptibility ratios were determined by the Wald method as mentioned in “Detection of Staphylococcal Enterotoxins” section.

### Detection of Staphylococcal Enterotoxins Genes

The isolates identified as *S. aureus* were screened for the presence of 11 main SE-encoding genes suspected to cause SFP outbreaks (*sea, seb, sec, sed, see, ser, seg, seh, sei, sej*, and *sep*) using two multiplex PCR assays according to the European Reference Laboratory (EURL) official method (Roussel et al., 2015). For *sea, seb, sec, sed, see*, and *ser* genes, the PCR amplification consisted of a reaction mixture containing 1X Supreme NZYTaq II 2 × Green Master Mix (NZYTech), 0.2 μM of each primer for *sea, seb, sec, ser*, 0.8 μM of each primer for *sed*, 0.6 μM of each primer for *see* and 2 μL of DNA template. Regarding the *seg, seh, sei, sej* and *sep* genes, the PCR amplification consisted of a reaction mixture containing 1X Supreme NZYTaq II 2 × Green Master Mix (NZYTech), 0.4 μM of each primer for *seh*, 0.6 μM of each primer for *seg*, 0.8 μM of each primer for *sei* and *sep*, 1.0 μM of each primer for *sej* and 2 μL of DNA template. Thermal cycling reaction conditions used were an initial denaturation at 95°C for 5 min, 35 cycles of denaturation at 94°C for 30 s, annealing for 30 s at 58°C and extension at 72°C for 30 s, and a final extension at 72°C for 5 min. Five reference *S. aureus* strains supplied by EURL CPS were used as positive controls (FRI137 for *sec*, *seg, seh, sei*; FRI361 for *seg, sei, sej, sec, sed, ser*; FRIS6 for *sea, seb*; FRI326 for *see*; and A900322 for *sei, seg, sep*). The PCR products were subjected to electrophoresis at 100 V for 1 h in a 2.5% (w/v) agarose gel, previous stained with GreenSafe Premium (NZYTech), and finally analyzed under UV light. CIs for prevalence of SE genes were determined by the Wald method as mentioned in “Detection of Staphylococcal Enterotoxins” section.

### Detection of Resistance Genes (*mecA* and *mecC*) and Other Virulence Genes (*pvl* and *tsst-1*)

*S. aureus* isolates were, also, characterized regarding the presence of methicillin resistance genes (*mecA* and *mecC*), as well as *pvl* and *tsst1* virulence genes. For the detection of *mecA*, *mecC* and *pvl* genes, a multiplex reaction was used as described by EURL protocol (Stegger et al., 2012). The PCR amplification consisted of a reaction mixture containing 1X Supreme NZYTaq II 2 × Green Master Mix (NZYTech), 0.2 μM of each primer for *mecA*, *mecC* and *pvl* and 2 μL of DNA template. The detection of the *tsst1* gene was performed using the primers described by Johnson et al. (1991) and a reaction mixture containing 1X Supreme NZYTaq II 2 × Green Master Mix (NZYTech), 0.5 μM of each primer for *tsst1* and 2 μL of DNA template. The thermal cycling reaction conditions used for both PCR were an initial denaturation at 95°C for 5 min, 35 cycles of denaturation at 94°C for 30 s, annealing for 30 s at 56°C for *mecA*, *mecC* and *pvl* genes or 54°C for *tsst1* gene, extension at 72°C for 30 s, and a final extension 72°C for 5 min. The PCR products were subjected to electrophoresis at 100 V for 1 h in a 2.0% (w/v) agarose gel, previous stained with GreenSafe Premium (NZYTech), and finally analyzed under UV light. All PCR reactions were performed in 20 μL and using MJ Mini Personal Thermal Cycler (Bio-Rad). CIs for the prevalence of virulence/resistance genes were determined by the Wald method as mentioned in “Detection of Staphylococcal Enterotoxins” section.

## Results

### Prevalence of *Staphylococcus aureus* and Staphylococcal Enterotoxins in Raw Milk

**FIGURE 1:**
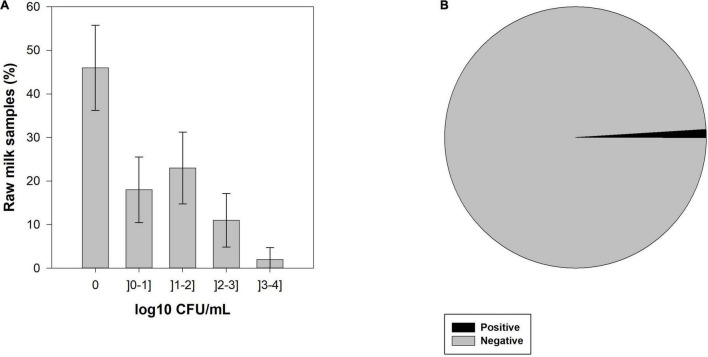
**(A)** Enumeration of bulk tank milk samples with coagulase-positive staphylococci (CPS) by colony forming units (CFU) categories and its 95% confidence interval (CI), and **(B)** prevalence of staphylococcal enterotoxins (SEs) in raw cow’s milk samples from bulk cooling tanks of 100 dairy farms located in the main dairy basin of mainland Portugal.

CPS were identified in 54.0% (95% CI: 41.6–66.4%) of raw milk samples and the number of colonies forming units (CFU) ranged between 0 and 3.4 log10 CFU.mL^–1^ (Figure 1A). One hundred and ten morphologically different CPS colonies were isolated, which means that in some cases more than one colony per raw milk sample was CPS characteristic and catalase positive. All isolates were tested for the *nuc* gene, and from the 110 CPS isolated, 104 isolates were confirmed as *S. aureus.* The remaining six CPS isolates were identified as *S. hyicus* (API^®^ Staph, bioMérieux). Of the 54 samples positive for CPS, *S. aureus* strains were identified in 53 (53.0%, 95% CI: 40.6–65.4%) of the samples.

From 100 bulk tank milk samples, only one was positive for the presence of SEA-SEE (Figure 1B). Interestingly, the SEs-positive sample was negative for the presence of CPS and, consequently, no *S. aureus* was isolated from that sample.

### *Spa* Typing Characterization

**FIGURE 2:**
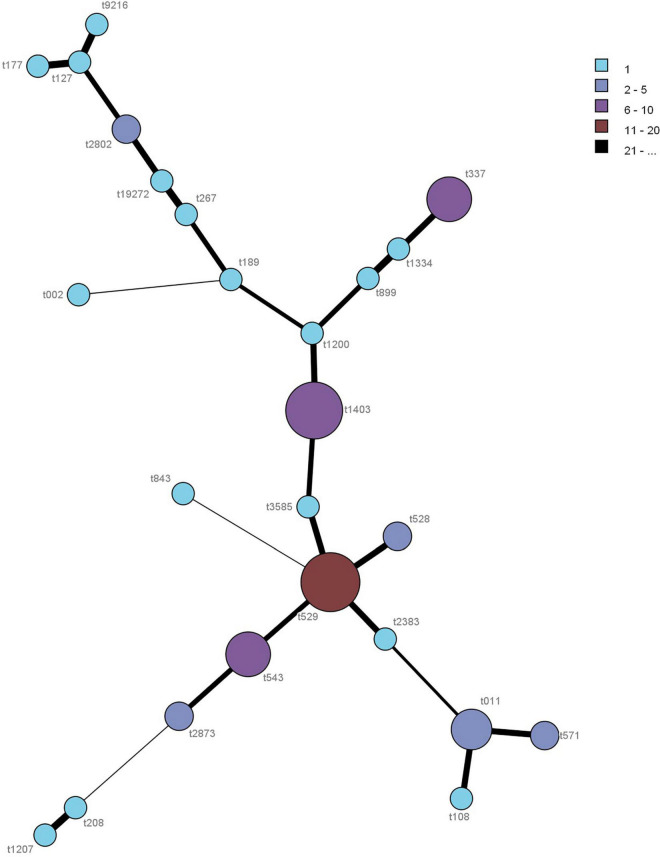
Minimum spanning tree of the spa typing for the 62 *S. aureus* isolates. Each spa type is depicted by a single node, with the size of the node representing the number of isolates associated with this spa type.

Among the 104 *S. aureus* isolates, 25 different *spa* types were detected. As the same *S. aureus* type was identified in different colonies from the same raw milk sample, only 62 *S. aureus* strains from different raw milk samples were considered as distinct and used for further characterization. In terms of prevalence, the most common spa type was t529 (17.7%, 95% CI: 8.2–27.3%) followed by t1403 (16.1%, 95% CI: 7.0–25.3%), t337 (9.7%, 95% CI: 2.3–17.0%), t543 (9.7%, 95% CI: 2.3–17.0%), and t011 (8.1%, 95% CI: 1.3–14.8%). Other spa types, such as t528, t571, t2802, and t2873 were associated with two (3.2%, 95% CI: 0.0–7.6%) distinct isolates, while t002, t108, t117, t127, t189, t208, t267, t843, t899, t1200, t1207, t1334, t2383, t3585, t9216, and t19272, were associated to one (1.6%, 95% CI: 0.0–4.7%) *S. aureus* isolate (Figure 2).

### Antimicrobial Susceptibility Test

**FIGURE 3:**
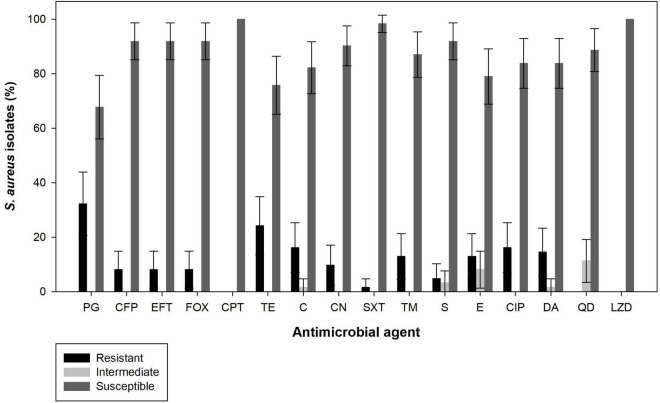
Antimicrobial susceptibility of *S. aureus* isolated from raw cow’s milk samples collected from bulk cooling tanks of 100 dairy farms located in the from main dairy basin in of mainland Portugal. PG, Penicillin G; FOX, cefoxitin; CPT, ceftaroline; CFP, cefoperazone; EFT, ceftiofur; TE, tetracycline; C, chloramphenicol; CN, gentamicin; SXT, trimethoprim-sulphamethoxazole; TM, trimethoprim; S, sulfonamides; E, erythromycin; CIP, ciprofloxacin; DA, clindamycin; QD, quinupristin-dalfopristin; LZD, linezolid.

The antimicrobial susceptibility of the 62 *S. aureus* isolates to 16 antimicrobial agents are shown in Figure 3. The highest resistance was observed for penicillin G (32.3%, 95% CI: 20.6–43.9%) followed by tetracycline (24.2%, 95% CI: 13.5–34.9%), chloramphenicol (16.1%, 95% CI: 7.0–25.3%) and ciprofloxacin (16.1%, 95% CI: 7.0–25.3%), and to a lesser extent clindamycin (14.5%, 95% CI: 5.7–23.3%), erythromycin (12.9%, 95% CI: 4.6–21.2%), trimethoprim (12.9%, 95% CI: 4.6–21.2%), gentamicin (9.7%, 95% CI: 2.3–17.0%), cefoperazone (8.1%, 95% CI: 1.3–14.8%), ceftiofur (8.1%, 95% CI: 1.3–14.8%), sulfonamides (4.8%, 95% CI: 0–10.2%), and trimethoprim–sulfamethoxazole (1.6%, 95% CI: 0–4.7%). No resistance was observed for ceftaroline, quinupristin-dalfopristin and linezolid. In addition, five isolates (8.1%, 95% CI: 1.3–14.8%) were identified as methicillin-resistant strains (cefoxitin resistance) and showed complete resistance to all other β-lactams tested, except to ceftaroline. Overall, 64.5% (95% CI: 52.6–76.4%) of the *S. aureus* isolates were susceptible to all antimicrobial agents tested and 35.5% (95% CI: 23.6–47.4%) of the *S. aureus* isolates were resistant to at least one of the antimicrobial agents tested, being that 9.7% (95% CI: 2.3–17.0%) were resistant to only one, 3.2% (95% CI: 0–7.6%) to two, and 22.6% (95% CI: 12.2–33.0%) were multi-drug resistant (resistant to three or more antimicrobial agents of different classes). Individual resistance profiles can be found in the Supplementary Table 2.

### Distribution of Genes Encoding Staphylococcal Enterotoxins

**FIGURE 4:**
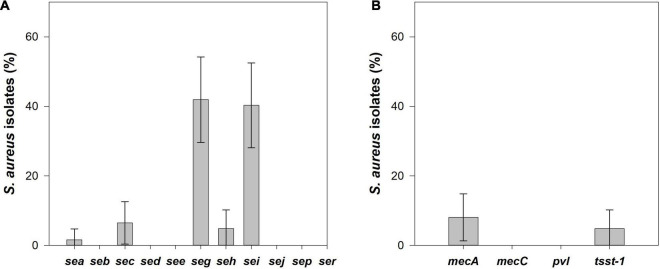
**(A)** Prevalence and its 95% confidence interval (CI) of enterotoxin coding genes, and **(B)** methicillin resistance genes, *mecA* and *mecC*, toxic shock syndrome toxin-1 (*tsst-1*) and Panton-Valentine leukocidin (*pvl*) genes in *S. aureus* isolated from raw cow’s milk samples collected from bulk cooling tanks of 100 dairy farms located in the main dairy basin of mainland Portugal.

Among the 62 *S. aureus* isolates, 46.8% (95% CI: 34.4–59.2%) harbored at least one of the SEs gene analyzed, 35.5% (95% CI: 23.6–47.4%) isolates had two SE genes and 4.8% (95% CI: 0.0–10.2%) isolates carried three SE genes (Figure 4A). From the 11 investigated SE genes, the *seg* was the most frequently detected (41.9%, 95% CI: 29.7–59.2%) followed by the *sei* (40.3%, 95% CI: 28.1–52.5%), *sec* (6.4%, 95% CI: 0.3–12.6%), *seh* (4.8%, 95% CI: 0–10.2%), and *sea* (1.6%, 95% CI: 0.0–4.7%). The genes *seg* and *sei* were always detected together (i.e., 40.3%, 95% CI: 28. 1–52.5%) of the *S. aureus* isolates, except one *S. aureus* isolate that was positive only for the *seg* gene. The gene *sec* was always detected in combination with *seg* and *sei*, and the isolate positive for *sea* was also positive for the *seh* gene. Moreover, *seh* gene was found alone in two isolates.

### Distribution of the Resistance Genes (*mecA* and *mecC*) and Virulence Genes (*pvl* and *tsst-1*)

From the 62 *S. aureus* isolates, 8.1% (95% CI: 1.3–14.8%), the same isolates that showed a cefoxitin resistance phenotype were confirmed as MRSA since they harbored the *mecA* gene. The *tsst1* gene was identified in 4.8% (95% CI: 0.0–10.2%) of the *S. aureus* isolates. No *pvl* or *mecC* genes were detected among the *S. aureus* analyzed (Figure 4B). MRSA isolates did not contain any genes encoding SEs or TSST-1, but all *tsst1*-positive isolates were enterotoxigenic *S. aureus* with the same SE gene pattern, *sec/seg/sei*.

### Genetic Patterns of *Staphylococcus aureus*

Based on the *spa* characterization and distribution of SEs-, resistance-, and other virulence-encoding genes, an analysis of the molecular patterns of *S. aureus* disseminated in raw cow’s milk was performed. Individual data for each isolate can be found in the Supplementary Table 3.

**FIGURE 5:**
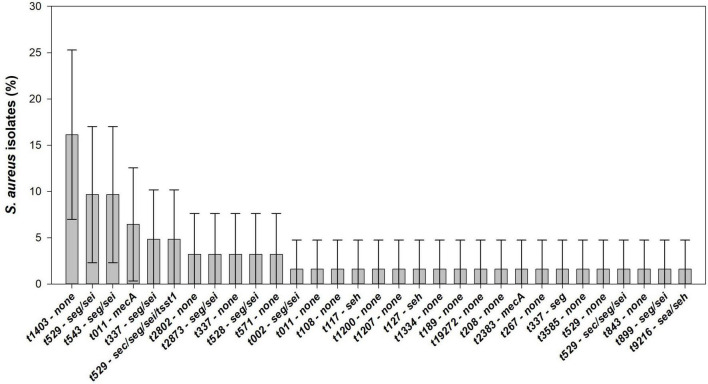
Distribution and its 95% confidence interval (CI) of virulence factors analyzed in *S. aureus* isolated from raw cow’s milk samples collected from bulk cooling tanks of 100 dairy farms located in the main dairy basin of mainland Portugal.

Seven genetic patterns can be identified: (1) *seg;* (2) *seh;* (3) *sea/seh;* (4) *seg/sei;* (5) *sec/seg/sei;* (6) *tsst-1/sec/seg/sei;* and (7) *mecA.* Furthermore, 45.2% (95% CI: 32.8–57.5%) of the *S. aureus* isolates did not present any of the virulence or resistance genes analyzed. Combining spa types and virulence/resistance determinants, 32 *S. aureus* patterns were detected among 62 isolates in raw milk samples from northern Portugal. Enterotoxigenic *S. aureus* are associated to spa types t002, t117, t127, t337, t528, t529, t543, t899, t2873, and t9216. Out of the five *mecA*-MRSA isolates, four were found to be t011 and one t2383. *S. aureus* t1403, t2802, t571, t108, t189. t208, t267, t843, t1200, t1207, t1334, and t19272 were exclusively associated with strains that did not contain any of the virulence/resistance genes evaluated. In total, *S. aureus* t1403-none (16.1%, 95% CI: 7.0–25.3%) is the predominant molecular pattern, followed by t529-*seg/sei* and t543-*seg/sei* (9.8%, 95% CI: 2.3–17.0%) (Figure 5).

## Discussion

The presence of *S. aureus* and its enterotoxins in raw milk can be a serious threat to public health given the importance of dairy products in human diet. Therefore, an estimation of the prevalence and genetic determinants of *S. aureus* is always important to implement rational mitigation strategies and avoid the dissemination of this pathogen through the food chain (Kadariya et al., 2014).

In this study, a prevalence of 53.0% of *S. aureus* was established in raw cow’s milk collected from the bulk cooling tanks of 100 dairy farms located in the main dairy region of mainland Portugal. To date, this is the first significant study of *S. aureus* in raw milk carried out in Portugal. A similar level of contamination (approximately 40–70%) in raw cow’s milk has been found in other countries such as Poland, Italy, New Zealand, Norway, India and China (Jørgensen et al., 2005b; Jakobsen et al., 2011; Hill et al., 2012; Bianchi et al., 2014; Sudhanthiramani et al., 2015; Rola et al., 2016; Liu et al., 2017; Wang et al., 2018; Kou et al., 2021). In fact, the presence of *S. aureus* in bulk tank milk is not surprising given that this species is ubiquitous in nature and one of the main causes of bovine mastitis. Even so, the EU regulation established a criterion for CPS in cheeses made from raw milk (m = 10^4^ CFU/g – M = 10^5^ CFU/g) during the manufacturing process. If values are higher than 10^5^ CFU/g cheese, the batch has to be tested for staphylococcal enterotoxin (EU Commission, 2007). In the present study, the CFU counts obtained on the CPS positive samples were all below the imposed limits. Low levels of CPS at the farms tanks are expected since milk storage is controlled at low temperatures (≈4°C) and for a short time after milking (12 – 24 h), as a way to prevent the growth of pathogens, including *S. aureus* (Owusu-Kwarteng et al., 2020).

Still, *S. aureus* presence in foods can represent a risk for human health since this pathogen produces an array of exoproteins with toxicological effects that are highly stable. Although the conditions used during the pasteurization process are suitable to destroy harmful microorganisms, they are not capable of eliminating some proteins, such as toxins. They will keep their activity in pasteurized milk, even at few micrograms, and be a serious threat to end consumers. From those toxins, SEs are the most relevant in the context of foodstuff. In fact, SEs are responsible for several SFP outbreaks reported every year worldwide (Argudín et al., 2010). Thus, their presence in foods is strictly prohibited in Europe (EU Commission, 2007; European Food Safety Authority and European Centre for Disease Prevention and Control, 2021a). In this study, one raw milk sample was positive for staphylococcal enterotoxins (SEA-SEE). Data on SEs in raw milk are scarce, as direct analysis on the raw material is out of scope of the European regulations. Although the presence of enterotoxins in raw milk has never been studied in Portugal, it has been found in cheeses during official controls, and also an outbreak affecting 13 persons due to an unknown food source (European Food Safety Authority [EFSA], 2019; European Food Safety Authority and European Centre for Disease Prevention and Control, 2021a). In Poland, the presence of SEs in raw milk was assessed without any positive result (Rola et al., 2016). Furthermore, SEs production usually requires the presence of *S. aureus* in high amounts, at least 5–6 log10 CFU.mL^–1^ (Argudín et al., 2010). None of the bulk tank milk samples analyzed had a level of contamination near or above this value, contributing to a lower probability of detection of SEs in the analyzed samples. Surprisingly, in the SE-positive sample found in this study no CPS was detected. Several hypotheses can be made to explain this result. Contamination of milk directly with enterotoxin from the udder of an animal is one of them. In fact, if *S. aureus* was in the udder of dairy cows with enough number of cells able to produce SEs, then enterotoxins could be transferred to the milk during the milking process, even after the bacteria have been eliminated (Rola et al., 2016). However, neither clinical nor subclinical mastitis were indicated in the animals from the dairy farm. Another possible explanation might be related with a dilution effect in the bulk milk, if only one or a few caws are infected. This dilution effect might affect more significantly culture techniques than SE determination, considering that the detection limit reported for VIDAS SET 2 is as low as 0.25 ng toxin per gram of food (Schultz et al., 2004). Enterotoxigenic coagulase-negative staphylococci (CNS) cannot be ruled out as several virulence determinants, usually associated with *S. aureus*, such as SE-encoding genes, have been detected in CNS genomes (Park et al., 2011; Podkowik et al., 2013) and different studies have identified several enterotoxigenic CNS species in dairy animals, capable of producing enterotoxins, predominantly SEC (Bergonier et al., 2003; Taponen et al., 2006; Ünal and Çinar, 2012). Since the methodology followed, in this study, for the isolation of *S. aureus* reject CNS strains, it is not possible to evaluate if CNS are responsible for the SE-positive sample. Finally, we should consider the possibility of a false positive result. Nonetheless, replicates were tested to confirm the sample result.

In recent years, the emergence of antimicrobial resistant strains, particularly MRSA, in livestock animals that is readily transferable to humans, has also become a growing public health concern (Sharma et al., 2018). In the present study, only 35.5% of the *S. aureus* isolates showed resistance to at least one antimicrobial agent. Similar results were reported on studies carry out in Italy (39.4%), Poland (23.0%) and China (38.5%) (Rola et al., 2015; Giacinti et al., 2017; Wang et al., 2018). Penicillin resistance was the most prevalent (32.3%) among the antimicrobial agents tested in accordance with previous studies in raw milk (Rola et al., 2015; Wang et al., 2018). Tetracycline, a broad-spectrum antimicrobial agent frequently used in the treatment of infections in cattle, was the second most prevalent antimicrobial resistance (24.2%), a level similar found to other studies (Liu et al., 2017). It was notable that ciprofloxacin- and chloramphenicol-resistant *S. aureus* were the third most frequently detected antimicrobial resistance. Ciprofloxacin is a third generation fluoroquinolone used at clinical level, while chloramphenicol is an antibiotic not authorized for use in food-producing animals in the European Union (Benford et al., 2014; Wang et al., 2018). Moreover, five isolates (8.1%) were identified as MRSA by antimicrobial susceptibility, revealing resistance to cefoxitin and all other β-lactams, except ceftaroline (Clinical and Laboratory Standards Institute [CLSI], 2020). Despite the low percentage of resistance observed, antibiotics should be used with caution in dairy animals because they may compromise the treatment of future infections. The presence of isolates with the MRSA phenotype is of major concern for human public health.

Although the presence of SEs was not detected in samples carrying *S. aureus*, enterotoxins’ production is still possible under appropriate environmental conditions (e.g., temperature) if the SE-encoding genes are part of the *S. aureus* genome. In our study, we have identified the presence of five different SE-encoding genes among 46.8% of *S. aureus* isolates, including two classical SE genes (*sea* and *sec*) and three non-classical SE genes (*seh, seg* and *sei*). Only five (8.1%) *S. aureus* isolates had classical SE genes (*sea-see*), although the literature suggested that about 95% of SFP outbreaks are caused by classical SEs (Argudín et al., 2010). Based on our results, the official method recommended for the detection of SEs in foodstuff does not reflect the complete diversity of enterotoxins found in nature. In fact, three of the SEs genes identified in this study, including the two most prevalent SEs (*seg* and *sei*), are not covered by ISO 19020:2017 methodology. The low prevalence of *sea-see* genes explains the low prevalence of SEA-SEE found in the bulk tank raw milk samples. Moreover, since the presence of non-classic SEs is not covered by the recommended methodologies, their prevalence may be underestimated. In Portugal, the characterization of *S. aureus* from a small number of raw milk samples had already verified the predominance of the *seg* and *sei* genes (Pereira et al., 2009). High prevalence of *seg* and *sei* was also observed in raw milk in Italy, and its co-existence in most isolates is also consistent with previous reports (Blaiotta et al., 2006; Bianchi et al., 2014; Johler et al., 2018). In fact, these two SE genes are typically located in tandem on the *ecg* locus and have already been implicated in scarlet fever, toxic shock and neonatal intractable diarrhea cases (Naik et al., 2008). However, these genes do not exist strictly together, as verified in one isolate of this study (only *seg*) and suggested by Jørgensen et al. (2005b). Other studies on raw milk have observed a higher prevalence of *sea-see* genes in Italy, Poland and China, yet most of them are in agreement with our study on the predominance of non-classic SE genes (Bianchi et al., 2014; Rola et al., 2016; Wu et al., 2018). Classical SE genes seems to be more prevalent in isolates of human and non-animal food origin than in animal food origin, such as *S. aureus* from raw milk (Chao et al., 2015). Furthermore, it should be mentioned that despite the prevalence of certain enterotoxin genes, they may have different genomic localization that may differentiate the virulence potential of the strains. Enterotoxins can be encoded in prophages, pathogenicity islands, genomic islands or plasmids, which can have different levels of regulation and expression of these virulence factors (Malachowa and Deleo, 2010).

Regarding the presence of resistance genes, the detection of *mecA* (8.1%) shows that raw cow’s milk can also be an antimicrobial resistance vehicle. The positive *mecA*-MRSA isolates correspond with the isolates classified as MRSA by the antimicrobial susceptibility test, demonstrating a complete correspondence between the genomic and phenotype results. The presence of MRSA strains have been reported in raw milk and dairy cattle in Europe (Tenhagen et al., 2014, 2018; Cortimiglia et al., 2016; Hansen et al., 2019; Schnitt et al., 2020; Lienen et al., 2021), but also in Algeria, Uganda, Brazil, and China (Asiimwe et al., 2017; Guimarães et al., 2017; Dai et al., 2019; Titouche et al., 2019). In Europe, MRSA prevalence in raw milk was 3–10%, which is in accordance with our results (Schnitt et al., 2020). In Portugal, *mecA*-MRSA strains have also been reported in bovine mastitis (Pereira et al., 2009), while *mecA* is also the predominant gene in most MRSA isolates worldwide. No association between MRSA strains and enterotoxigenic determinants was found in our analysis. In contrast, TSST-1 genetic determinant was found in enterotoxigenic isolates (*sec/sec/sei*-positive). Such relation may result in an increase in the toxigenic consequences of these strains. No *pvl* or *mecC* genes were detected, consistent with previous studies demonstrating the low prevalence of these genes among *S. aureus* isolated from dairy products (van Duijkeren et al., 2014; Johler et al., 2018). However, three isolates were identified as spa types (t843 and t528) commonly associated to CC130, the frequent genetic background of *mecC* (Bortolami et al., 2017). Given the epidemiological relevance of this genetic determinant of MRSA, the negative result for the presence of the *mecC* gene was confirmed. Furthermore, these isolates revealed a complete susceptibility to the tested antimicrobials, including cefoxitin (*mecC*-positive strains are typically cefoxitin resistant).

Regarding the diversity of *S. aureus*, we have identified 25 spa types among the 62 *S. aureus* isolates from the 100 bulk tank milk samples. Wang et al. (2018) found seven *spa* types of *S. aureus* in 96 isolates from raw milk collected on 2 dairy farms in China. The lower diversity obtained on that study might be due to the limited number of farms enrolled. Furthermore, the most predominant types observed in our study, t529 and t1403, differed from the t127 observed in China. A better correspondence is observed when compared to studies in European countries. Higher diversity and t529 and t1403 types were also predominant in raw milk and bovine isolates in Denmark and Switzerland (Johler et al., 2011; Ronco et al., 2018). Most of the other spa types detected in our study were also associated with bovine *S. aureus* from different European countries (Boss et al., 2016). Thus, the diversity of *S. aureus* found in raw milk in Portugal seems to be in line with that found in the rest of Europe, suggesting a geographic predominance of some spa types. In terms of human health, spa types t002 and t127, detected in one isolate each, are frequently observed in human invasive infections (Grundmann et al., 2010). Combining spa typing with virulence factors, 32 distinct *S. aureus* types can be identified in this study. Some spa types were exclusively associated to enterotoxigenic *S. aureus*, mainly t002, t117, t127, t528, t543, t899, t2873, and t9216. In contrast, *S. aureus* t1403 was also exclusively associated with the absence of virulence/resistance factors. The MRSA-t011 type have recently emerged in European countries, such as Germany and Denmark, associated with livestock-associated MRSA (LA-MRSA) strains including from raw milk isolates (van Duijkeren et al., 2014; Tenhagen et al., 2018; Hansen et al., 2019; Schnitt et al., 2020; Lienen et al., 2021). Accordingly, our results support that this trend is also present in Portugal. MRSA-t2383 type is a rare relative of t011 and was never reported in raw milk or dairy cattle, but in human and other animals as pigs and seabream (Fanoy et al., 2009; Salgueiro et al., 2020). Thus, spa type/virulence/resistance factors can be a good way to assess the variation in *S. aureus* diversity over time. In most instances, a *spa* types are highly associated with a specific multilocus sequence type (MLST), which is usually related with a specific clonal lineage (Tegegne et al., 2019). As an example, identified spa-types t002, t108, t117, t529, t1334, and t1207 are usually assigned to either sequence type (ST) ST151 or ST504, both belonging to clonal complex (CC) CC70, while t1403 is usually associated with ST97-CC97 or ST133-CC133. In the case of MRSA isolates, the spa-types here identified are usually assigned to ST398-CC398, which is the prevalent LA-MRSA in Europe, occasionally involved in human disease (Bortolami et al., 2017). Overall, most of the ST/CC associated with the spa-types identified in this study are commonly associated with livestock infection or carriage, especially of bovine origin (Smith et al., 2005; Hasman et al., 2010; Boss et al., 2016).

## Conclusion

In conclusion, the high prevalence of enterotoxigenic *S. aureus* and the detection of MRSA strains in raw cow’s milk collected from bulk cooling tanks on dairy farms from the main dairy region of mainland Portugal is of particular concern. Furthermore, a high diversity of *S. aureus* was found in a relatively small geographical area, however, most with genomic lineages associated with livestock infection or carriage. This study also points out the predominance of SE-encoding genes that are not currently covered by the gold standard methodology (ISO 19020:2017) applied in the control of food samples. The higher prevalence of non-classical SEs, mainly SEG, SEH and SEI, should not be ignored, as these have been implicated in food poisoning outbreaks (Jørgensen et al., 2005a; Tang et al., 2011; Ciupescu et al., 2018; Hait et al., 2018). Commercial tests with proven effectiveness for the non-classic SEs are available on the market, which may facilitate their integration in food safety control standard for SEs analysis of foods (Hait et al., 2018). As SFP outbreaks linked to raw cow’s milk consumption and raw milk products have been increasing, the presented data on the characterization of *S. aureus* and its virulence determinants are important to improve risk assessment and develop solutions to limit the dissemination of this pathogen in Portugal.

## Data Availability Statement

The original contributions presented in the study are included in the article/Supplementary Material, further inquiries can be directed to the corresponding author.

## Author Contributions

RO, EP, GA, NA, and CA designed the experiments. RO performed the experiments. RO, EP, and CA analyzed the data. All authors contributed to the writing of the manuscript.

## Conflict of Interest

The authors declare that the research was conducted in the absence of any commercial or financial relationships that could be construed as a potential conflict of interest.

## Publisher’s Note

All claims expressed in this article are solely those of the authors and do not necessarily represent those of their affiliated organizations, or those of the publisher, the editors and the reviewers. Any product that may be evaluated in this article, or claim that may be made by its manufacturer, is not guaranteed or endorsed by the publisher.
