# Supplementary material for: Prevalence and Diversity of Staphylococcus aureus and Staphylococcal Enterotoxins in Raw Milk From Northern Portugal
Source: Front Microbiol. 2022 Mar 22;13:846653. doi: 10.3389/fmicb.2022.846653 (PMC8981150; doi:10.3389/fmicb.2022.846653)
Supplement: Supplementary file 1 [file Data_Sheet_1.PDF]

Supplementary Table S1. Individual data from collected samples and from the screening for coagulase-positive *staphylococci* and enterotoxins in raw cow's milk samples from bulk cooling tanks of 100 dairy farms located in the main dairy basin of mainland Portugal.

ID code	No. of animals	Horizontal method for the enumeration of coagulase-positive staphylococci - ISO/FDIS 6888-2<br>Method using Baird Parker agar medium with Rabbit Plasma Fibrinogen (RPF)										Detection of staphylococcal enterotoxins types SEA to SEE
		Typical coagulase positive colonies	No. Typical Colonies									
			1E-01		1E-02		1E-03		Total cfu/mL	log10		
			Rep1	Rep2	Rep1	Rep2	Rep1	Rep2				Vidas SET2
E1	40	No	0	0	0	0	-	-	-	-		Negative
E2	120	No	0	0	0	0	-	-	-	-		Negative
E3	85	Yes	4	3	1	0	-	-	3,5E+01	1,5		Negative
E4	95	Yes	1	1	0	0	-	-	1,0E+01	1,0		Negative
E5	50	Yes	44	37	6	4	-	-	4,1E+02	2,6		Negative
E6	44	Yes	3	2	0	0	-	-	2,3E+01	1,4		Negative
E7	100	No	0	0	0	0	-	-	-	-		Negative
E8	88	No	0	0	0	0	-	-	-	-		Negative
E9	120	Yes	22	25	3	1	-	-	2,3E+02	2,4		Negative
E10	100	No	0	0	0	0	-	-	-	-		Negative
E11	45	No	0	0	0	0	-	-	-	-		Negative
E12	125	No	0	0	0	0	-	-	-	-		Negative
E13	100	Yes	17	15	3	4	-	-	1,8E+02	2,2		Negative
E14	94	Yes	3	2	0	1	-	-	2,5E+01	1,4		Negative
E15	50	Yes	4	3	1	0	-	-	3,5E+01	1,5		Negative
E16	40	No	0	0	0	0	-	-	-	-		Negative
E17	75	Yes	15	11	2	3	-	-	1,4E+02	2,1		Negative
E18	100	Yes	6	5	2	1	-	-	5,5E+01	1,7		Negative
E19	80	No	0	0	0	0	-	-	-	-		Negative

E20	97	No	0	0	0	0	-	-	-	-	Negative
E21	68	Yes	1	0	0	0	-	-	5,0E+00	0,7	Negative
E22	30	No	0	0	0	0	-	-	-	-	Negative
E23	32	No	0	0	0	0	-	-	-	-	Negative
E24	130	Yes	2	0	0	0	-	-	1,0E+01	1,0	Negative
E25	24	Yes	2	0	0	0	-	-	1,0E+01	1,0	Negative
E26	50	No	0	0	0	0	-	-	-	-	Negative
E27	12	No	0	0	0	0	-	-	-	-	Positive
E28	65	No	0	0	0	0	-	-	-	-	Negative
E29	100	Yes	32	27	5	5	-	-	3,1E+02	2,5	Negative
E30	50	No	0	0	0	0	-	-	-	-	Negative
E31	20	No	0	0	0	0	-	-	-	-	Negative
E32	100	No	0	0	0	0	-	-	-	-	Negative
E33	150	Yes	12	20	1	3	-	-	1,6E+02	2,2	Negative
E34	50	Yes	2	0	0	0	-	-	1,0E+01	1,0	Negative
E35	55	No	0	0	0	0	-	-	-	-	Negative
E36	35	No	0	0	0	0	-	-	-	-	Negative
E37	40	No	0	0	0	0	-	-	-	-	Negative
E38	85	Yes	3	8	1	0	-	-	5,5E+01	1,7	Negative
E39	45	No	0	0	0	0	-	-	-	-	Negative
E40	45	Yes	1	4	0	1	-	-	2,5E+01	1,4	Negative
E41	30	Yes	0	1	0	0	-	-	5,0E+00	0,7	Negative
E42	35	No	0	0	0	0	-	-	-	-	Negative
E43	65	Yes	1	1	0	0	-	-	1,0E+01	1,0	Negative
E44	105	Yes	3	0	0	0	-	-	1,5E+01	1,2	Negative
E45	45	Yes	2	0	0	0	-	-	1,0E+01	1,0	Negative
E46	30	Yes	2	0	0	0	-	-	1,0E+01	1,0	Negative
E47	80	Yes	3	2	0	0	-	-	2,5E+01	1,4	Negative

E48	24	Yes	1	0	0	0	-	-	5,0E+00	0,7	Negative
E49	70	Yes	1	0	0	0	-	-	5,0E+00	0,7	Negative
E50	87	No	0	0	0	0	-	-	-	-	Negative
E51	50	Yes	186	169	22	23	1	0	1,8E+03	3,3	Negative
E52	60	No	0	0	0	0	-	-	-	-	Negative
E53	60	Yes	46	41	5	2	-	-	4,3E+02	2,6	Negative
E54	30	Yes	21	30	6	2	-	-	2,7E+02	2,4	Negative
E55	40	Yes	9	5	2	0	-	-	7,0E+01	1,8	Negative
E56	61	Yes	6	4	1	2	-	-	5,0E+01	1,7	Negative
E57	82	No	0	0	0	0	-	-	-	-	Negative
E58	110	Yes	2	2	1	0	-	-	2,0E+01	1,3	Negative
E59	48	No	0	0	0	0	-	-	-	-	Negative
E60	80	Yes	1	0	0	0	-	-	5,0E+00	0,7	Negative
E61	85	No	0	0	0	0	-	-	-	-	Negative
E62	71	No	0	0	0	0	-	-	-	-	Negative
E63	60	No	0	0	0	0	-	-	-	-	Negative
E64	70	Yes	3	2	0	0	-	-	2,5E+01	1,4	Negative
E65	110	Yes	2	0	1	0	-	-	1,0E+01	1,0	Negative
E66	46	No	0	0	0	0	-	-	-	-	Negative
E67	110	Yes	2	4	0	0	-	-	3,0E+01	1,5	Negative
E68	135	No	0	0	0	0	-	-	-	-	Negative
E69	40	No	0	0	0	0	-	-	-	-	Negative
E70	108	Yes	3	5	1	0	-	-	4,0E+01	1,6	Negative
E71	80	No	0	0	0	0	-	-	-	-	Negative
E72	100	Yes	1	0	0	0			5,0E+00	0,7	Negative
E73	40	No	0	0	0	0	-	-	-	-	Negative
E74	46	Yes	7	3	0	0	-	-	5,0E+01	1,7	Negative
E75	75	Yes	4	1	0	0	-	-	2,5E+01	1,4	Negative

E76	174	Yes	1	0	0	0	-	-	5,0E+00	0,7	Negative
E77	80	Yes	4	2	1	0	-	-	3,0E+01	1,5	Negative
E78	80	Yes	8	11	2	0	-	-	9,5E+01	2,0	Negative
E79	25	No	0	0	0	0	-	-	-	-	Negative
E80	50	No	0	0	0	0	-	-	-	-	Negative
E81	110	No	0	0	0	0	-	-	-	-	Negative
E82	100	Yes	1	4	1	0	-	-	2,5E+01	1,4	Negative
E83	50	Yes	3	2	1	0	-	-	2,5E+01	1,4	Negative
E84	88	Yes	1	0	0	0	-	-	5,0E+00	0,7	Negative
E85	266	Yes	18	21	4	7	-	-	2,3E+02	2,4	Negative
E86	70	No	0	0	0	0	-	-	-	-	Negative
E87	50	No	0	0	0	0	-	-	-	-	Negative
E88	36	No	0	0	0	0	-	-	-	-	Negative
E89	55	Yes	1	0	0	0	-	-	5,0E+00	0,7	Negative
E90	90	Yes	9	9	1	1	-	-	9,0E+01	2,0	Negative
E91	50	No	0	0	0	0	-	-	-	-	Negative
E92	65	Yes	6	9	2	0	-	-	7,5E+01	1,9	Negative
E93	76	Yes	13	11	5	5	-	-	1,2E+02	2,1	Negative
E94	116	No	0	0	0	0	-	-	-	-	Negative
E95	130	Yes	248	276	43	47	-	-	2,8E+03	3,4	Negative
E96	104	Yes	1	0	0	0	-	-	5,0E+00	0,7	Negative
E97	150	No	0	0	0	0	-	-	-	-	Negative
E98	170	No	0	0	0	0	-	-	-	-	Negative
E99	80	Yes	13	13	4	2			1,3E+02	2,1	Negative
E100	75	No	0	0	0	0	-	-	-	-	Negative

Supplementary Table S2. Individual resistance profiles of *S. aureus* isolated from raw cow's milk samples from bulk cooling tanks of 100 dairy farms located in the main dairy basin of mainland Portugal.

Antimicrobial agent		Code/Concentration	Sensible ≥ (mm)	Intermediate (mm)	Resistant ≤ (mm)
Penicillinase-labile penicillins	Penicillin	PG 10	29	-	28
Cephalosporin III	Cefoperazone	CFP 30	23	18-22	17
	Ceftiofur	EFT 30	21	18-20	17
Cephamycin	Cefoxitin	FOX 30	22	-	21
Cephems	Ceftaroline	CPT 5	20	19-20	17
Tetracyclines	Tetracycline	TE 30	19	18-15	14
Phenicols	Chloramphenicol	C 30	18	13-17	12
Aminoglycosides	Gentamicin	CN 10	15	13-14	12
Folate pathway antagonists	Trimethoprim-sulfamethoxazole	SXT 25	16	15-11	10
	Sulfonamides	S 300	17	16-13	12
	Trimethoprim	TM 5	16	15-11	10
Macrolides	Erythromycin	E 15	23	22-14	13
Fluoroquinolones	Ciprofloxacin	CIP 5	21	20-16	15
Lincosamides	Clindamycin	DA 2	21	20-15	14
Streptogramins	Quinupristin-dalfopristin	QD 15	19	16-18	15
Oxazolidinones	Linezolid	LZD 30	21	-	20

Isolate no.	PG 10	CFP 30	EFT 30	FOX 30	CPT 5	TE 30	C 30	CN 10	SXT 25	TM 5	S 300	E 15	CIP 5	DA 2	QD 15	LZD 30
E3S3	S	S	S	S	S	S	S	S	S	S	S	S	S	S	S	S
E4S4	S	S	S	S	S	S	S	S	S	S	S	S	S	S	S	S
E5S5	S	S	S	S	S	S	S	S	S	S	S	S	S	S	S	S
E5S6	S	S	S	S	S	S	S	S	S	S	S	S	S	S	S	S
E6S9	S	S	S	S	S	S	S	S	S	S	S	S	S	S	S	S

E6S10	S	S	S	S	S	S	S	S	S	S	S	S	S	S	S	S
E9S11	S	S	S	S	S	S	S	S	S	S	S	S	S	S	S	S
E9S12	S	S	S	S	S	S	S	S	S	S	I	S	S	S	S	S
E13S14	R	R	R	R	S	R	S	S	S	S	S	S	R	S	S	S
E14S17	R	S	S	S	S	S	S	S	S	S	S	S	S	S	S	S
E15S20	R	S	S	S	S	R	R	R	S	R	S	R	R	R	I	S
E17S23	S	S	S	S	S	S	S	S	S	S	S	S	S	S	S	S
E21S29	R	S	S	S	S	S	S	R	S	S	S	S	S	S	S	S
E24S30	S	S	S	S	S	S	S	S	S	S	S	S	S	S	S	S
E25S32	R	S	S	S	S	S	S	S	S	S	I	R	S	S	S	S
E29S34	S	S	S	S	S	S	S	S	S	S	S	S	S	S	S	S
E33S37	R	S	S	S	S	R	R	S	S	R	S	S	R	R	S	S
E33S39	S	S	S	S	S	S	S	S	S	S	S	S	S	S	S	S
E34S40	R	S	S	S	S	R	R	R	S	R	S	R	R	R	I	S
E34S41	R	S	S	S	S	R	R	R	S	R	S	S	R	R	S	S
E38S42	R	S	S	S	S	S	S	S	S	S	S	S	S	S	S	S
E40S44	S	S	S	S	S	S	S	S	S	S	S	S	S	S	S	S
E41S47	S	S	S	S	S	S	S	S	S	S	S	S	S	S	S	S
E43S48	S	S	S	S	S	S	S	S	S	S	S	S	S	S	S	S
E44S49	S	S	S	S	S	S	S	S	S	S	S	S	S	S	S	S
E45S52	R	S	S	S	S	R	R	S	S	R	R	R	R	R	I	S
E46S53	S	S	S	S	S	S	S	S	S	S	S	S	S	S	S	S
E47S54	R	S	S	S	S	R	R	R	S	R	S	R	R	R	I	S
E48S55	S	S	S	S	S	S	S	S	S	S	S	S	S	S	S	S
E49S56	S	S	S	S	S	S	S	S	S	S	S	S	S	S	S	S
E51S59	S	S	S	S	S	S	S	S	S	S	S	S	S	S	S	S
E53S61	S	S	S	S	S	S	S	S	S	S	S	S	S	S	S	S

E54S62	S	S	S	S	S	S	S	S	S	S	S	S	S	S	S	S
E54S63	S	S	S	S	S	S	S	S	S	S	S	I	S	S	S	S
E55S64	S	S	S	S	S	S	S	S	S	S	S	S	S	S	S	S
E56S66	R	S	S	S	S	S	S	S	S	S	S	I	S	S	S	S
E56S67	R	S	S	S	S	R	R	S	R	R	R	R	R	R	I	S
E58S68	R	R	R	R	S	R	R	S	S	S	S	I	R	R	I	S
E60S70	S	S	S	S	S	S	S	S	S	S	S	S	S	S	S	S
E64S71	S	S	S	S	S	S	S	S	S	S	S	S	S	S	S	S
E65S73	S	S	S	S	S	S	S	S	S	S	S	S	S	I	S	S
E67S75	S	S	S	S	S	S	S	S	S	S	S	S	S	S	S	S
E70S77	S	S	S	S	S	S	S	S	S	S	S	S	S	S	S	S
E72S79	S	S	S	S	S	S	S	S	S	S	S	S	S	S	S	S
E74S80	S	S	S	S	S	S	S	S	S	S	S	S	S	S	S	S
E75S83	S	S	S	S	S	S	S	S	S	S	S	S	S	S	S	S
E76S84	R	S	S	S	S	R	R	S	S	S	S	S	S	S	S	S
E77S85	R	R	R	R	S	R	S	S	S	S	S	I	S	S	S	S
E77S86	S	S	S	S	S	S	S	S	S	S	S	S	S	S	S	S
E78S89	R	S	S	S	S	R	R	R	S	R	R	R	R	R	I	S
E82S92	S	S	S	S	S	S	I	S	S	S	S	S	S	S	S	S
E83S94	S	S	S	S	S	S	S	S	S	S	S	R	S	S	S	S
E84S95	S	S	S	S	S	S	S	S	S	S	S	S	S	S	S	S
E85S96	S	S	S	S	S	S	S	S	S	S	S	S	S	S	S	S
E85S98	S	S	S	S	S	S	S	S	S	S	S	S	S	S	S	S
E89S99	S	S	S	S	S	S	S	S	S	S	S	S	S	S	S	S
E90S100	R	S	S	S	S	S	S	S	S	S	S	S	S	S	S	S
E92S102	S	S	S	S	S	S	S	S	S	S	S	S	S	S	S	S
E93S105	S	S	S	S	S	R	S	S	S	S	S	S	S	S	S	S

E95S107	S	S	S	S	S	S	S	S	S	S	S	S	S	S	S	S
E96S110	R	R	R	R	S	R	S	S	S	S	S	I	S	S	S	S
E99S111	R	R	R	R	S	R	S	S	S	S	S	S	S	S	S	S

Supplementary Table S3. Individual data of virulence/resistance factors analyzed in *S. aureus* isolated from raw cow's milk samples from bulk cooling tanks of 100 dairy farms located in the main dairy basin of mainland Portugal.

**Sample**		**Agar-based detection**		**PCR-based detection**							**MLST**	
**Isolate no.**	**Microorganism**	**Baird-Parker agar Coagulase ativity**	**Catalase test**	***nucA***	***mecA***	***mecC***	***pvl***	***tst-1***	***se genes***	***spa typing***	***Molecular pattern***	***Inferred ST/CC***
E3S3	*S. aureus*	+	+	+	-	-	-	-		t267	t267-none	ST97 (CC97)
E4S4	*S. aureus*	+	+	+	-	-	-	-	seg, sei	t529	t529-seg/sei	ST151/ST504 (CC705)
E5S5	*S. aureus*	+	+	+	-	-	-	-		t1403	t1403-none	ST97 (CC97) or ST133 (CC133)
E5S6	*S. aureus*	+	+	+	-	-	-	-	sec, seg, sei	t529	t529-sec/seg/sei	ST151/ST504 (CC705)
E6S9	*S. aureus*	+	+	+	-	-	-	-	seg, sei	t543	t543-seg/sei	ST479 (CC479)
E6S10	*S. aureus*	+	+	+	-	-	-	-		t2802	t2802-none	Not found
E9S11	*S. aureus*	+	+	+	-	-	-	-		t1403	t1403-none	ST97 (CC97) or ST133 (CC133)
E9S12	*S. aureus*	+	+	+	-	-	-	+	sec, seg, sei	t529	t529-sec/seg/sei/tsst1	ST151/ST504 (CC705)
E13S14	*S. aureus*	+	+	+	+	-	-	-		t011	t011-mecA	ST398 (CC398)
E14S17	*S. aureus*	+	+	+	-	-	-	-		t208	t208-none	ST49
E15S20	*S. aureus*	+	+	+	-	-	-	-	seg, sei	t337	t337-seg/sei	ST20 (CC29)
E17S23	*S. aureus*	+	+	+	-	-	-	-	seh	t127	t127-seh	ST1 (CC1)
E21S29	*S. aureus*	+	+	+	-	-	-	-		t011	t011-none	ST398 (CC398)
E24S30	*S. aureus*	+	+	+	-	-	-	-	seg, sei	t543	t543-seg/sei	ST479 (CC479)
E25S32	*S. aureus*	+	+	+	-	-	-	-		t2802	t2802-none	Not found
E29S34	*S. aureus*	+	+	+	-	-	-	-	seg, sei	t543	t543-seg/sei	ST479 (CC479)
E33S37	*S. aureus*	+	+	+	-	-	-	-	seg, sei	t337	t337-seg/sei	ST20 (CC29)

E33S39	*S. aureus*	+	+	+	-	-	-	-		t3585	t3585-none	Not found
E34S40	*S. aureus*	+	+	+	-	-	-	-	seg, sei	t337	t337-seg/sei	ST20 (CC29)
E34S41	*S. aureus*	+	+	+	-	-	-	-	seg	t337	t337-seg	ST20 (CC29)
E38S42	*S. aureus*	+	+	+	-	-	-	-		t1200	t1200-none	ST9 (CC9)
E40S44	*S. aureus*	+	+	+	-	-	-	-		t1403	t1403-none	ST97 (CC97) or ST133 (CC133)
E41S47	*S. aureus*	+	+	+	-	-	-	-		t529	t529-none	ST151/ST504 (CC705)
E43S48	*S. aureus*	+	+	+	-	-	-	+	sec, seg, sei	t529	t529-sec/seg/sei/tsst1	ST151/ST504 (CC705)
E44S49	*S. aureus*	+	+	+	-	-	-	-	seg, sei	t529	t529-seg/sei	ST151/ST504 (CC705)
E45S52	*S. aureus*	+	+	+	-	-	-	-		t337	t337-none	ST20 (CC29)
E46S53	*S. aureus*	+	+	+	-	-	-	-		t1403	t1403-none	ST97 (CC97) or ST133 (CC133)
E47S54	*S. aureus*	+	+	+	-	-	-	-		t337	t337-none	ST20 (CC29)
E48S55	*S. aureus*	+	+	+	-	-	-	-	seg, sei	t543	t543-seg/sei	ST479 (CC479)
E49S56	*S. aureus*	+	+	+	-	-	-	-	seg, sei	t528	t528-seg/sei	ST130 (CC130)
E51S59	*S. aureus*	+	+	+	-	-	-	-	seh	t117	t117-seh	ST504 (CC705)
E53S61	*S. aureus*	+	+	+	-	-	-	-	seg, sei	t2873	t2873-seg/sei	ST479 (CC479)
E54S62	*S. aureus*	+	+	+	-	-	-	-		t571	t571-none	ST398 (CC398)
E54S63	*S. aureus*	+	+	+	-	-	-	-		t843	t843-none	ST130 (CC130)
E55S64	*S. aureus*	+	+	+	-	-	-	-	seg, sei	t002	t002-seg/sei	ST504 (CC705)
E56S66	*S. aureus*	+	+	+	-	-	-	-	seg, sei	t543	t543-seg/sei	ST479 (CC479)
E56S67	*S. aureus*	+	+	+	-	-	-	-	seg, sei	t899	t899-seg/sei	ST398 (CC398) or ST9 (CC9)
E58S68	*S. aureus*	+	+	+	+	-	-	-		t011	t011-mecA	ST398 (CC398)
E60S70	*S. aureus*	+	+	+	-	-	-	-	seg, sei	t543	t543-seg/sei	ST479 (CC479)
E64S71	*S. aureus*	+	+	+	-	-	-	-		t1403	t1403-none	ST97 (CC97) or ST133 (CC133)
E65S73	*S. aureus*	+	+	+	-	-	-	-	seg, sei	t529	t529-seg/sei	ST151/ST504 (CC705)
E67S75	*S. aureus*	+	+	+	-	-	-	-		t1403	t1403-none	ST97 (CC97) or ST133 (CC133)
E70S77	*S. aureus*	+	+	+	-	-	-	-	seg, sei	t529	t529-seg/sei	ST151/ST504 (CC705)
E72S79	*S. aureus*	+	+	+	-	-	-	-		t1403	t1403-none	ST97 (CC97) or ST133 (CC133)

E74S80	*S. aureus*	+	+	+	-	-	-	-		t1403	t1403-none	ST97 (CC97) or ST133 (CC133)
E75S83	*S. aureus*	+	+	+	-	-	-	-	seg, sei	t528	t528-seg/sei	ST130 (CC130)
E76S84	*S. aureus*	+	+	+	-	-	-	-		t108	t108-none	ST504 (CC705)
E77S85	*S. aureus*	+	+	+	+	-	-	-		t2383	t2383-mecA	ST398 (CC398)
E77S86	*S. aureus*	+	+	+	-	-	-	-	seg, sei	t2873	t2873-seg/sei	ST479 (CC479)
E78S89	*S. aureus*	+	+	+	-	-	-	-		t1334	t1334-none	ST504 (CC705)
E82S92	*S. aureus*	+	+	+	-	-	-	-		t1403	t1403-none	ST97 (CC97) or ST133 (CC133)
E83S94	*S. aureus*	+	+	+	-	-	-	-		t571	t571-none	ST398 (CC398)
E84S95	*S. aureus*	+	+	+	-	-	-	-	seg, sei	t529	t529-seg/sei	ST151/ST504 (CC705)
E85S96	*S. aureus*	+	+	+	-	-	-	-	seg, sei	t529	t529-seg/sei	ST151/ST504 (CC705)
E85S98	*S. aureus*	+	+	+	-	-	-	-		t1207	t1207-none	ST504 (CC705)
E89S99	*S. aureus*	+	+	+	-	-	-	-	sea, seh	t9216	t9216-sea/seh	Not found
E90S100	*S. aureus*	+	+	+	-	-	-	-		t189	t189-none	ST188 (CC188)
E92S102	*S. aureus*	+	+	+	-	-	-	-		t1403	t1403-none	ST97 (CC97) or ST133 (CC133)
E93S105	*S. aureus*	+	+	+	-	-	-	+	sec, seg, sei	t529	t529-sec/seg/sei/tsst1	ST151/ST504 (CC705)
E95S107	*S. aureus*	+	+	+	-	-	-	-		t19272	t19272-none	Not found
E96S110	*S. aureus*	+	+	+	+	-	-	-		t011	t011-mecA	ST398 (CC398)
E99S111	*S. aureus*	+	+	+	+	-	-	-		t011	t011-mecA	ST398 (CC398)
